# Loss, shame and secrecy in women’s experiences of a vulval skin condition: A qualitative study

**DOI:** 10.1177/13634593241271041

**Published:** 2024-08-11

**Authors:** Sophie Rees, Susanne Arnold

**Affiliations:** University of Bristol Medical School, UK; University of Warwick Medical School, UK

**Keywords:** dermatology, gender, Lichen sclerosus, stigma, vulval disease

## Abstract

Vulval lichen sclerosus (LS) is a chronic dermatological condition affecting the anogenital area, causing intense itching, pain and bleeding. It can change the terrain of the vulva, causing loss of vulval anatomy and altered texture and appearance of the skin. There has been little research into how women experience the materialities of a dermatological vulval disease. We aimed to understand experiences of living with LS, using a feminist lens to examine the influence of societal attitudes towards women’s bodies and the vulva. We conducted qualitative interviews with 20 women with vulval LS, taking a critical feminist grounded theory approach. While we found that women’s experiences of vulval LS symptoms was normalised as a part of womanhood, there was a silencing of speech about the vulva generally, and vulval symptoms more specifically. This caused profound shame and loneliness, and was a barrier to disclosing and seeking help for vulval symptoms, leading to delayed diagnosis and disease progression. Loss of vulval architecture resulted in a loss of (feminine) self and the sense of a body which was whole.

## Introduction

Chronic dermatological conditions which affect the vulva can have a profound impact on quality of life and identity, but they have received almost no consideration in the social science literature. The little attention paid to vulval skin diseases such as lichen sclerosus, lichen planus, vulval eczema, and even vulval cancer, reflects the invisibility of this highly signified part of the body in wider society. While awareness and understanding of diseases such as breast and cervical cancer have improved, conditions of the vulva represent a largely unexplored frontier in the sociology of health, and a taboo subject in everyday life. Without an understanding of the experience of vulval conditions, we cannot address impact on quality of life or identify ways to improve the experiences of people with the condition.

In this paper we aim to contribute the voices of women with vulval lichen sclerosus (LS) to the literature, shedding light on this rarely-explored experience. There are two main strands to our analysis in this paper: feminist analysis of the phenomenology of shame, and historical and contemporary analyses of women and/in pain. We take a critical feminist approach, drawing on theories of shame, gender and the abject, to explore how sociocultural attitudes towards the vulva and women’s health issues, shape the experience of a vulval skin condition.

## Vulval lichen sclerosus

Lichen sclerosus (LS) is a chronic inflammatory vulval skin condition typically causing intense pruritus (itching) and fissures (small splits in the skin) making toileting, sexual activity and moving or even sitting painful. In research measuring impact on quality of life, LS has been found to significantly affect functional activities, sexual activity, as well as self-esteem and confidence (Lansdorp et al., 2013; Van De Nieuwenhof et al., 2010). In a 2015 survey, one in five women with a vulval condition had considered suicide or self-harm (British Association of Dermatologists, 2015). According to clinical guidelines, LS is usually manageable with an ultrapotent topical corticosteroid ointment and moisturising emollients (Lewis et al., 2018). Symptoms typically abate with the correct treatment, becoming a lifelong flaring and remitting condition.

While the experience of vulvodynia (chronic genital pain) has received some attention (Labuski, 2015; Shallcross et al., 2019) the particular materialities of a dermatological vulval condition have been overlooked. LS changes the very terrain of the vulva. It can cause visible and structural changes to the genital skin: the vulval skin becomes white in pallor, and thin and fragile in some places or thickened and ‘scarred’ in others. Untreated, LS may cause progressive loss of vulval architecture: in a process known as ‘fusing’, the vaginal entrance may narrow, and the clitoris may be ‘buried’ as the prepuce and labial skin fuse together. This can happen early in the disease and in some cases is irreversible without surgery. LS is also considered a risk factor for cancer: although vulval cancer is rare, rates are 20 times higher in those with LS (Lewis et al., 2018). Treatment is critical to preventing disease progression, but diagnosis is often very delayed, reported to be between 5 and 15 years from symptom onset (Cooper et al., 2004). Understanding the experience of LS may help us identify how to improve access to diagnosis and treatment, as well as patients’ quality of life.

## Shame, women and the vulva

In this analysis we draw on feminist scholars of the phenomenology of shame who have highlighted the long association between women, shame and the body (Bartky, 2015; Dolezal, 2015a). If the body is understood as constitutive of the self (Merleau-Ponty, 1962), a diseased body can be experienced as a failure of the self, and exposes one as vulnerable and flawed. Body shame may occur when a person feels that their body does not meet sociocultural notions of normality, desirability, or acceptability, such as when a person falls ill (Dolezal, 2015b). An unwell or diseased body, then, can be ‘a powerful source and site of shame’ (Dolezal, 2015b).

However, shame can also be understood as inherent to the experience of womanhood in a sexist society, connected to the requirement to keep the body under control in response to the male gaze (Dolezal, 2015a). We argue that women experience shame simply in relation to *having* a vulva. Female genitalia are stigmatised and poorly understood. Labuski refers to the concept of genital or vulval ‘dis-ease’ to describe the cultural taboo, derision and silence of the vulva (Labuski, 2015). The use of the term ‘pudendum’ to refer to the vulva was recently removed from the *Terminologia Anatomica* (the international standard for human anatomical terminology), because the Latin root of the word (*pudere*) means ‘to be ashamed’ (Draper, 2021; Neumann et al., 2020). Although as others point out, ‘changing one word will not undo generations of implicit bias and institutional oppression’ (Draper, 2021: 318). Women internalise genital stigma, resulting in adverse health outcomes (Holland et al., 2020). The media, especially pornographic media, presents an unrealistic ideal of women’s genitalia, and female genital diversity has been pathologised by the plastic surgery industry, leading to a rise in women and girls seeking or receiving labiaplasty (Braun, 2019; Chibnall et al., 2020).

A meta-ethnography of the qualitative literature on experiences of vulvodynia (Shallcross et al., 2019) concluded that shame was a key dimension of the experience of vulvodynia, and that social narratives about womanhood, sexuality and femininity were a driving force of their negative experiences. For example, the ‘coital imperative’ is a narrative which equates penile penetration with ‘real’ sex (McPhillips et al., 2001), and women experienced shame as they were unable to fulfil this imperative, and felt like ‘unreal women’ (Kaler, 2006).

According to Kristeva (1982) the abject is that which:

disturbs identity, system and order. What does not respect boundaries, positions, rules. The in-between, the ambiguous, the composite. (Kristeva, 1982: 4)

The vulva itself can be understood as abject, transgressing the boundary of internal/external. Its folds can be experienced as mysterious and secretive, and it needs to be ‘opened’ in order to be fully explored, yet it also forms part of the external terrain of our bodies. It is the pathway through which bodily fluids exit, such as urine and menstrual blood, and through which other body parts or entities may pass for example, during sex, or tampon insertion. It is elusive, with dark corners and folds, requiring mirrors and a level of mobility to visually self-examine or self-explore. Knowledge about the anatomy of the vulva amongst the general population, and also medical professionals, is limited (Preti et al., 2021; Woodgate and Hayes, 2019), and addressing this could help demystify the vulva.

A diseased vulva, then, may cause women to experience shame both because of the failure of the self to be healthy, and because the disease affects a body part that is rendered abject, and which women are conditioned to be ashamed of having at all. Using Goffman’s work on stigma, having a vulval disease (which is largely invisible) may result in women being ‘discreditable’, leading to anxieties around disclosure, and subsequent shame and loneliness (Goffman, 1963). Shame is a frequent feature and a powerful force in the medical encounter (Dolezal, 2015b), especially when the illness or body part in question is socially sensitive or intimate in nature (Consedine et al., 2007). It has been described as a ‘potent treatment barrier’ (Saraiya and Lopez-Castro, 2016), and empirical evidence indicates that people may ignore serious symptoms in order to avoid the shame that would arise from revealing their symptoms to others, even health professionals (Consedine et al., 2007).

There has been minimal research into how women experience their vulva in the presence of a dermatological vulval disease, and how the phenomenology of shame and notions of the abject may illuminate their experiences, including of seeking help for symptoms.

## Women and pain

This second strand of our analysis relates to societal beliefs about women in pain. Feminist historical analyses explain that the medical institution’s approach to women and pain or illness has changed over time. In the mid-19th century, a pale and sickly woman was believed to be more feminine (Munch, 2004). A new condition ‘hysteria’ was created at the start of the 20th century, the supposed ‘wandering’ of the uterus to other parts of a woman’s body, causing various symptoms and irrational behaviour (Munch, 2004). The idea of women as emotionally fragile remained in medical thought, and stereotypes endure about women’s pain as exaggerated, untroublesome, psychosomatic and even fabricated (Fischer, 2004; Scott et al., 2022).

Chronic pain conditions disproportionately affect women and those assigned female at birth (Bailey and Bernstein, 2013), yet women’s accounts of pain are often doubted, disbelieved and undermined (Bailey and Bernstein, 2013; Scott et al., 2022), and they are less likely to receive appropriate treatment for pain (Hoffmann and Tarzian, 2001). In particular, women with gynaecological conditions struggle for recognition and treatment. For example, women with endometriosis wait on average six and a half years for diagnosis (Nnoaham et al., 2011) and gaining recognition for sexual pain is a challenge (Braksmajer, 2018). Healthcare professionals dismiss, discount or minimise women’s experiences of pain during consultations (Scott et al., 2022; Shallcross et al., 2019; Ussher, 2013), and gynaecological procedures (Harrison et al., 2020; Maguire et al., 2014). Research indicates that menstrual and gynaecological pain is normalised as simply part of womanhood through discourses during childhood and young adulthood (Scott et al., 2022), and this may affect women’s experiences of seeking and accessing diagnosis and treatment for vulval LS.

The dismissal and normalisation of women’s pain further cements the stigma and shame around talking about genital pain and a disinclination to discuss genital health issues on the part of healthcare professionals (Nguyen et al., 2013). Time from reported symptoms to diagnosis for LS may range from 5 to 15 years (Krapf et al., 2020), which amounts to many years of suffering with symptoms before (hopefully) receiving the correct treatment. For a progressive condition such as vulval LS, this delay can result in profound loss and irreversible damage to the structure of their vulva, and the onset of vulval cancer. Delayed access to treatment may therefore have dire consequences for women with LS.

In this paper we explore women’s embodied experiences of vulval LS, applying a sociological and explicitly feminist lens to their accounts. We examine how societal beliefs about women’s pain shaped the experience of living with vulval LS.

## Methods

### Methodology

We adopted a critical feminist approach, which involves problematising the status quo and interrogating historical, cultural and contextual conditions (Scott et al., 2022). A critical feminist grounded theory approach involves using the constant comparison analysis method to examine how underlying assumptions about meaning in everyday life produce experience and oppression, with a focus on generating knowledge which can effect social change (Kushner and Morrow, 2003). This approach brings an explicitly feminist and critical lens to grounded theory. This strategy enabled us to situate participants’ experiences of living with a vulval disease in the context of wider social structures and relations of power, and to ground our theorising in participants’ accounts.

### Patient advisory panel

Our patient advisory panel was made up of three women with vulval LS who were involved from the study’s inception. It includes one of the organisers of a large online support group and awareness page. We met with them on a number of occasions to discuss: topic guide; recruitment; approach to distress; and our evolving interpretation. Our discussions with them aided our analysis and development of this paper. Specifically, while they emphasised the challenges of living with LS symptoms, they encouraged us to focus on the silence around the condition as they felt this had a major impact on their everyday life and emotional wellbeing.

### Setting and participants

The data collection took place in England and Wales in 2020–2021. Inclusion criteria were: ⩾18 years old; a self-reported diagnosis of vulval LS; able to converse in English. We recruited via online support and education communities, and posters in community centres. We wanted to access individuals who would be engaged with the topic and be able to provide rich data about the experience of living with LS.

We recognise that individuals who do not identify as women can also experience vulval LS, such as trans men and non-binary people. We kept our recruitment materials gender-neutral, and shared an invitation to participate in the study via an active trans organisation on social media. However, we did not attract any expressions of interest from trans or non-binary individuals, therefore we have used the term ‘woman’ throughout this paper to refer to our participants and past research. Trans people experience institutional erasure of their experiences when accessing healthcare, and the absence of their representation is a limitation of this study, and is a gap in the literature on the experiences of vulval and other pelvic or genital conditions (Bauer et al., 2009; Scott et al., 2022).

Individuals who expressed an interest were sent a participant information leaflet and given the opportunity to ask questions. If willing to participate, respondents were asked for a small amount of demographic data to facilitate purposive sampling using a maximum variation approach in terms of age, gender, ethnicity and time since diagnosis.

### Data collection

Due to COVID-19 we offered telephone or virtual video call interviews using Microsoft Teams instead of face-to-face interviews. Interviews were audio-recorded with participants’ consent. Verbal consent was audio-recorded prior to the interview and stored separately. Interviews were semi-structured. We used a flexible topic guide and began with a broad question: ‘Would you like to start by talking about when you first noticed a problem?’ to encourage participants to give their accounts in their own words. The topic guide was developed with our patient advisory panel. We also made field notes to capture additional information and document emerging ideas.

### Data analysis

We drew on Charmaz’s guidelines for social constructionist grounded theory data collection and constant comparative analysis (Charmaz, 2014). Pseudonymised and checked transcripts were analysed in QSR NVivo 12. Analysis began during data collection, using a constant comparative method. This involved initially coding the interviews, developing themes from this coding, comparing new nodes within themes against one another to understand how they provide new dimensions to the theme, and returning to raw data to consider how it might add further to our evolving themes and conceptual claims. Both authors independently coded early transcripts to discuss the coding approach and initial interpretations. Transcripts were coded using descriptive labels to summarise ‘what is going on’ in excerpts of data, creating numerous codes per interview, which were then grouped under umbrella nodes (Charmaz, 2014). Through this continuous iterative process, categories and themes were developed in order to create an interpretation of the data. We reviewed our developing analysis as it progressed, including with our patient advisory panel, to develop key and overarching themes.

### Ethical approval

The study was approved by the University of Warwick BSREC 02/20-21. We had a protocol in place to deal with participant distress during interviews. After every interview the authors debriefed and discussed any difficult parts of the interview.

## Results

### Participants

A total of 71 women expressed an interest, 41 provided information for purposive sampling, and 20 interviews were completed between November 2020 and May 2021. Mean duration was 60 minutes (range 45–80 minutes). All participants identified as female, and the majority (80%) were white (Table 1). We did not receive expressions of interest from anyone who did not identify as a cis woman. Mean age was 53 years (range 23–72), mean number of years since diagnosis was six (range <1 to <23). Pseudonyms are used in the following Results section.

**Table 1.** Participant characteristics.

Characteristic	Number
Age	
20–29 years	2
30–39 years	2
40–49 years	3
50–59 years	4
60–69 years	7
70–79 years	2
Ethnic identity	
Asian or Asian British	0
Black or African or Caribbean or Black British	2
Mixed or Multiple Ethnic Group	2
White	16
Years since diagnosis	
<1	3
1–4	8
5–9	5
10–14	2
15–20	1
>20	1

### Feeling ‘unclean’

Vulval LS is not contagious or caused by poor hygiene practices, yet women commonly described feeling ‘dirty’ or ‘unclean’, invoking notions of infection or contagion. They associated vulval disease with sexually transmitted diseases, resulting in shame and self-blame.

Women, we, we think if we’ve got something, you know, sort of, wrong down there that it makes us feel dirty. You know that we’ve done something wrong. – Yvonne

I thought it maybe, it was linked to maybe I should have a had a wash three times a day or something like that. – Camille

I actually felt very ashamed of myself. I thought it had to be down to me. I think I still carry a kind of shame about it. . .fault in some way. – Catherine

Visible indications of their condition sometimes stopped them from certain activities or even from going out at all, especially during a flare-up.

I was staying at home because when I was wearing moisturisers and oils and things they were seeping through my clothes and I was, I was getting quite embarrassed by it. I felt quite ashamed and, sort of, broken. – Grace

Sometimes I feel them *[leggings]* rubbing when I’m running on the treadmill. And I have to keep, sort of, pulling them away from my, my vulva. Which, you know, if I was outside and other people saw me, I don’t think it would look very nice. – Sarah

A lot of the time I wasn’t able to wear *[underwear]*. Then I couldn’t really go out. – Fiona

Visible events like scratching or seeping moisturisers brought the vulva into public view and consciousness, and made women feel ashamed or embarrassed. LS was something to be ashamed of due to its location, and something which many initially blamed themselves for. They saw themselves as other, positioned outside of the norm and potentially polluting. Some women physically separated themselves from others during a flare to avoid the stigma attached to the visible signs of their condition.

### Disclosing to others

Women identified a silence around the vulva, creating shame and preventing women from speaking about their experiences.

It’s all very hush-hush . . .It’s not talked about. Using the correct terminology around the vulva and the vagina, it’s like they’re dirty words. – Patricia

It’s not the kind of thing you discuss in polite society with your friends around, around a table. – Yvonne

I just feel I’m gonna get judged. – Paula

One participant described experiencing revulsion from potential sexual partners when she disclosed her diagnosis.

They [dates] would act like it was something horrible or they didn’t wanna catch it, even though I’d said to them, ‘You can’t catch it’. – Madeleine

Being a disease located in the genitals made LS potentially ‘discrediting’ (Goffman, 1963), and women anticipated, or experienced, judgement from others.

The lack of knowledge about LS amongst the general population meant they had to go into intimate detail.

Because it’s, yeah, on the genitals and it’s not well-known, it can be awkward. – Madeleine

If you have cancer, everyone knows what cancer is . . . I get really bad splits and sores and white patches and ulcers and, you know, it’s really hard to explain to somebody without them, like, them getting embarrassed as well as me getting embarrassed. – Janice

Giving voice to their vulva by describing their symptoms to others was experienced as taboo, making it more difficult for them to share.

The loneliness was considered one of the hardest dimensions of living with LS, and addressing this was an urgent concern for many of our participants

LS is about loneliness . . . Dealing with it alone. That is what LS is about. – Camille

I know there are other people like me who have to really change how their life is because of it. And secretly. And we do it secretly ’cause you don’t tell anyone. – Sandra

Half a dozen of my friends might have it. But we don’t talk about it. – Patricia

LS was experienced as a lonely disease, and most of our participants had only spoken to their partner or spouse about it. Because of the lack of knowledge amongst others about the condition, they would have to share specific details about how their vulva looked and felt, and this was taboo. They feared, or experienced, others’ revulsion towards them if they disclosed, and they worried that others would blame them for their condition and assume it was due to poor hygiene practices or sexual behaviour.

### Seeking help for LS symptoms

Women felt it was taboo to talk or, as Paige says below, even *think* about their vulva.

I’ve never been told that it’s okay to have a vagina [sic] and that it’s, and that it’s completely normal to look at it and, you know, like see it for what it is, and that it’s, you know, that, that it’s an organ in, in your body that’s, you know, not a bad thing . . . You have to keep everything hidden and you just don’t talk about it, don’t look at it, you know, don’t even, you know, like *think* about it. – Paige [Participant’s emphasis]

Paige and other participants described delaying seeking help because of the acute shame they felt about disclosing their symptoms, even to healthcare professionals.

And it, it just started getting progressively worse and worse and worse but I, I just dealt with it because I mean it’s, you know it’s quite an embarrassing thing to bring up, or at least for me to, you know, bring up with my doctor about it. – Paige

When it’s your health and especially when it’s an embarrass, embarrassing topic, I just find it excruciating to talk about. – Amanda

Seeking help required participants to overcome profound embarrassment to speak of their symptoms and of their vulva, but when they did eventually disclose to a healthcare professional, they were often left feeling dismissed by healthcare professionals.

The doctors were basically telling me it was, kind of, in my head. . .I think it’s already hard enough to bring it up with your doctor. But when you get dismissed it’s just, it really gets you down. – Madeleine

He was really, really dismissive and on the, on the border of being rude really. Basically said, ‘Well, why don’t you just go to the chemist and buy your own thrush treatment?’ And, and didn’t examine me or, or do anything really. So, at that point I was quite upset and felt abandoned, and nobody cared. – Hazel

Interviewees felt exposed and vulnerable during consultations and vulval examinations, and if their concerns were not addressed sensitively, they felt dismissed and degraded.

I remember driving home and crying that it was just, like, such a horrible experience to have to show an intimate area of myself to someone who was quite dismissive and, sort of, derogatory in the way that they spoke to me. – Amanda

And it’s difficult to talk about your, your symptoms or your worries or anything when you, your legs are akimbo and you’ve got, you know, your bits showing up on a screen and, and someone talking to you over the top of your, your bits, kind of thing. – Carol

Women avoided seeking help for as long as they could, often using over-the-counter thrush medication, to avoid the shame of verbalising their symptoms or speaking about their vulva to another person. However, the symptoms eventually became so overwhelming they had to seek help, but healthcare professionals were often dismissive of women’s concerns, leaving them feeling abandoned and humiliated.

### Vulval symptoms as part of womanhood

All participants were unaware of vulval skin conditions before learning they had LS, and rationalised their symptoms, often as a yeast infection (thrush) or simply as part of ageing or menopause. Pain or itching was often normalised, and things ‘going wrong’ with the vulva or vagina was considered an expected part of womanhood.

When I was 16 and it, I tore and things I just thought, ‘Oh, that, that must just be what sex is like’. – Grace

Being sore down there is just one of those things that women have. Over our lives, we, we have different things that happen in that area. – Fiona

I know obviously that, that ladies when we have children, we change colour. And I thought, ‘Oh, maybe it’s because, because I’m starting to go through the menopause’. – Yvonne

Healthcare professionals also sometimes perpetuated the normalisation of the women’s pain or other symptoms, repeatedly treating for thrush, even despite negative swabs, or suggesting the explanation was hormone- or age-related.

It was misdiagnosed as thrush for about four years . . . They wouldn’t look outside the box to see what the problem was. – Janice

I went to the GP, and she just said, ‘Oh it’s post-menopausal’. – Elizabeth

They often described feeling angry about how they were treated, linking it to attitudes towards women in healthcare in general.

Women are being failed . . . We are either ignored or, you know, we’re still classed as being hysterical, or that we don’t know our own bodies. – Yvonne

It still is an enshrined attitude that women don’t know themselves and they’re just making it up. – Teresa

Also, age discrimination, you know older women ‘Oh well you have to accept that some things will happen in life, you know there will be aches and pains’ I went through that when I was looking after my mother and I was, you know incensed on her behalf. And now I’m incensed on my own behalf. – Elizabeth

That a disease existed which could cause such profound loss for women, yet the public and healthcare professionals held such poor knowledge and understanding of, shocked and angered the women.

### Loss of (feminine) self through a progressive disease

The final part of our analysis explores the embodied experience of material changes due to LS in a context of shame and silence around the vulva. A number of participants in our study suffered changes to their normal vulval architecture, and this was experienced as a profound loss of the wholeness of their body.

I feel like I’ve got a gap down there, I feel like I’ve got a hole . . . . Sometimes when I’m walking along it feels, it just feels like there’s a gap there. – Sarah

You realise that bits of you are disappearing. Like I no longer have what you call it, a clitoris . . . It’s like, it’s like a boat without a sail. – Camille

The women connected their vulval anatomy to their sense of womanhood, and often felt ‘degendered’ or ‘defeminised’ (Kaler, 2006) through this loss of anatomy.

You lose architecture, all of that which is part of how you define yourself as a woman, suddenly begins to be eroded. – Elizabeth

I don’t feel like, it looks like a woman’s labia, labia anymore, it looks like a child’s. You, you know, how little girls that don’t have the full definition. – Sarah

I said to my husband, ‘I completely feel not like a woman. I feel, like, I’m, like, this invisibility. Like I’ve got this invisibility cloak’. . . . I just felt like I was nothing. – Sandra

Through the loss of anatomy, the women entered an abject liminal state: no longer fully a woman, not a child, a less-than woman. The women experienced a loss of (feminine) self through the material loss of their vulval anatomy.

Women struggled to describe this experience, which they felt was unlike anything they had encountered. A number of them described it as their body turning on itself, and destroying or ‘eating’ itself.

It’s like my vagina is eating itself. That’s just, that’s not a thing that happens to any part, any other part of my body. – Tanya

I just had, sort of, mental images of the whole of my genitals disappearing and being eaten by my own body. – Hazel

My skin’s just slowly eating itself. – Janice

Women drew direct links between the lack of support from healthcare professionals, and their lost vulval anatomy.

My clitoris got more and more buried . . . If I’d started the steroids earlier, that might not have happened. – Hazel

I’ve lost a lot of structure down there now. I think a lot of it went in the first four years over not having the correct treatment. Once it’s gone there’s no going back from that.” – Janice

From their perspective, this profound sense of loss was caused by delayed access to diagnosis and treatment, which was in turn caused by the normalisation and dismissal of women’s pain in medical encounters, as well as the shame and silence which prevented them from seeking help earlier.

## Discussion

Previous research about women’s experiences of LS has been conducted in the health sciences paradigm (Bentham et al., 2021; Brauer et al., 2015, 2016; Wehbe-Alamah et al., 2012) and this is the first study of the lived experience of a vulval dermatological condition to draw on social theories such as stigma and the abject, and apply an explicitly feminist lens to understand women’s experiences of LS. Our analysis highlights how agency was constrained, and loss was produced, both through the material effects of LS but also the shame and secrecy around the vulva and women’s bodies. We found that women experienced shame simply in relation to *having* a vulva, let alone a vulval *disease*. Experiencing vulval symptoms meant they had to confront a part of their body which they felt embarrassed even thinking about, let alone speaking about. Such internalised stigma has been reported by others as a barrier to good health outcomes (Holland et al., 2020). Societal norms constrained talk about the vulva, and women felt unable to disclose their illness to others, including healthcare professionals. Vulval pain was normalised as part of being a woman and having a vulva. This delayed access to diagnosis and treatment, resulting in material loss of their vulva, damaging their sense of a whole, feminine self. Their accounts demonstrated how loss was produced through the impact of attitudes towards women’s bodies in pain, secrecy around the vulva and lack of knowledge about vulval skin conditions.

Similar to a recent meta-ethnography on vulvodynia (Shallcross et al., 2019), we found that women felt ‘defeminised’ or ‘degendered’ by their vulval condition. In vulvodynia, this was due to being unable to fulfil the ‘coital imperative’ (McPhillips et al., 2001). Participants in this study experienced the same issues, but with a further dimension – they faced a physical loss of their vulval anatomy, which was described as a loss of the completeness of their body, and of their (feminine) self. The burial of their clitoris under their labial skin was experienced as a literal gap or hole: the terrain of their bodies had been changed. Through this loss of anatomy, the women faced another layer of abjection and were positioned in a liminal state: no longer fully a woman, not a child, a less-than woman. The double forces of abjection operate here – while the vulva transgresses in its very being, the attention required to the vulva in the case of LS operates as a further potent disruption, one which ‘disturbs identity, system and order’ (Kristeva, 1982). Brauer et al. (2015) found that a major driver for women seeking vulval surgery to address anatomical changes due to LS were the desire to be a ‘normal’ woman. While none of our participants discussed having vulval surgery, our study demonstrates the connection women made between the physical terrain of their vulva and their womanhood. Gender was accomplished in part through the material body, and the erosion of vulval architecture was experienced as a literal erosion of womanhood. The description from one of our participants of ‘like a boat without a sail’ expressed the physical loss of her clitoris, the loss of sexual pleasure and sensation she had previously enjoyed, as well as her lost womanhood.

Like any chronic illness, LS attracts a stigma. As a largely invisible disease, it could be concealed most of the time, making women with LS potentially ‘discreditable’ if they were to disclose their diagnosis, rather than visibly ‘discredited’ (Goffman, 1963; Harris, 2009). However, this concealment involved work, and women sometimes avoided going out in public during a flare. The abject body is also that which cannot be easily contained, and visibly seeping moisturisers and scratching were seen as something to be hidden and kept out of public view, lest it bring into consciousness the existence of their vulva and vulval disease. Many of the women described feeling ‘unclean’ or ‘dirty’, invoking notions of infection and contagion. Women worried that others would view them as dirty or unclean, and as having a sexually transmitted disease. They saw themselves as other, positioned outside of the norm, defeminised and potentially polluting. The chronic illness sufferer can be understood as abject, as they may appear well but experience symptoms which compromise their quality of life, disturbing and disrespecting order and boundaries between the ‘well’ and the ‘unwell’ (Harris, 2009; Sontag, 2001).

Early in this paper we stated that LS is usually manageable with treatment. While true in theory, our study shows that in practice there are multiple hurdles to overcome to gain diagnosis and appropriate treatment. These hurdles can be understood as largely structural, informed by the position of women in society, and by attitudes towards women’s bodies and the vulva. They first had to overcome the internalised normalisation of women’s genital symptoms. It was viewed as normal to experience changes ‘down there’ as a woman. Previous research has found that discourses encountered in early life which normalise menstrual and sexual pain prevent women from seeking help for painful pelvic conditions later in life (Scott et al., 2022). Our study indicates that genital symptoms or vulval changes in general are normalised, especially in menopause. While the recent increased awareness and openness about menopause is undoubtedly a positive development, without an accompanying increase in awareness of LS, women and health professionals may continue to mistakenly think that their LS symptoms are due to menopause. As remedies for urogenital atrophy become available over the counter, women may continue to delay seeking help from medical professionals for their LS symptoms.

Once women decided to seek help, they then had to deal with the shame and humiliation of speaking of their vulva, which even in a clinical setting resulted in a profound sense of shame. As Lazare (1987) describes:

Once in the examining room, patients must reveal personal information often about their weaknesses, expose their bodies, place themselves in undignified postures, and accept handling of their bodies including intrusions into orifices. (Lazare, 1987: 1655)

The women in our study were forced into this vulnerable position repeatedly, due to poor responses from healthcare professionals who repeatedly misdiagnosed their symptoms as thrush or simply part of menopause. Even when not examined, women felt vulnerable, laid bare, through the act of having to disclose their symptoms, especially when healthcare professionals discount or dismiss their experiences. Previous research (Bentham et al., 2021; Wehbe-Alamah et al., 2012) using analysis of online forums found that women expressed frustration over delayed diagnosis. A recent study found that those with vulval symptoms were at higher risk of adverse health experiences than those with non-genital dermatology symptoms, and that adverse health experiences increased time to diagnosis (Rivera et al., 2024). In our data, women drew connections to broader issues of being ignored or dismissed by medical professionals. It is well established that sexism exists in the medical profession and emerges during patient-doctor interactions, causing under-treatment and misdiagnosis of health problems in women, from chronic fatigue syndrome to cardiac arrest (Elderkin-Thompson and Waitzkin, 1999; Hamberg et al., 2004; Hoffmann and Tarzian, 2001; Miaskowski, 1999; Munch, 2004; Richman et al., 2000). In our data, ageism intersected with gender, as (peri)menopausal women were told that their LS symptoms were just part of menopause and to be expected.

These obstacles resulted in loss for the women in our study, in terms of an erosion of their feminine identity and sense of bodily wholeness (Merleau-Ponty, 1962). Vulval LS is a progressive disease, and participants connected the delay in diagnosis and treatment to the physical loss of their vulval anatomy, and attributed this delay to shame, stigma and a poor understanding of vulval disease. Narratives which normalise living with vulval pain or itching, but silence about female sexuality and the vulva, delayed help-seeking, and prevented healthcare professionals from taking women’s experiences seriously and making a prompt diagnosis. In this way, we argue that social narratives about women contributed to the material and emotional losses experienced by the women. Tackling the stigma and taboo around talking about the vulva, and improving knowledge of vulval disease amongst the general population and healthcare professionals, could reduce the impact of LS on women’s lives in future.

## Conclusion

In this paper we have begun to address a gap in the sociological literature about experiences of vulval skin conditions, as a first step towards identifying potential ways to improve the lives of those with LS. The vulva is a highly signified body part, and vulval disease causes women to experience an erosion of their (feminine) identity, and their sense of their body as whole and complete. Silence and secrecy around the vulva caused poor understanding of LS, and restricted agency in terms of being able to disclose their experience, resulting in loneliness and isolation for the women in our study. Women linked delayed diagnosis and treatment, to attitudes towards women’s pain as normalised or minimised. Loss, in terms of physical anatomy, and loss of self, was therefore produced through societal attitudes towards women’s bodies, and shame and stigma about the vulva.
